# Diversity of *Methylobacterium* spp. in the Rice of the Vietnamese Mekong Delta

**DOI:** 10.1264/jsme2.ME19111

**Published:** 2020-01-23

**Authors:** Khoa Lai, Ngoc Thai Nguyen, Hiroki Miwa, Michiko Yasuda, Hiep Huu Nguyen, Shin Okazaki

**Affiliations:** 1 United Graduate School of Agricultural Science, Tokyo University of Agriculture and Technology, Saiwaicho 3–5–8, Fuchu, Tokyo 183–8509, Japan; 2 National Agro-Forestry-Fisheries Quality Assurance Department branch 4, 271—To Ngoc Van St, Linh Dong ward, Thu Duc district, Ho Chi Minh City, Vietnam; 3 Graduate School of Agriculture, Tokyo University of Agriculture and Technology, Saiwaicho 3–5–8, Fuchu, Tokyo 183–8509, Japan; 4 Department of Microbial Biotechnology, Biotechnology Research and Development Institute, Cantho University, II—St 3/2, Ninh Kieu ward, Cantho City, Vietnam

**Keywords:** *Methylobacterium*, rice, plant growth promotion, disease, Mekong delta

## Abstract

The Vietnamese Mekong delta is one of the largest rice-producing areas globally. *Methylobacterium* spp. are persistent colonizers of the rice plant and exert beneficial effects on plant growth and health. Sixty-one *Methylobacterium* strains belonging to seven species were predominantly isolated from the phyllosphere of rice cultivated in six Mekong delta provinces. Inoculation tests revealed that some strains exhibited plant growth-promoting activity. Moreover, three strains possessed the novel characteristics of inducing leaf bleaching and killing rice seedlings. These results revealed the complex diversity of *Methylobacterium* in Mekong delta rice and that healthy and productive rice cultivation requires a proper balance of *Methylobacterium*.

The plant phyllosphere harbors diverse microbial communities and bacteria constitute the dominant population (Kembel *et al.*, 2014). The mechanisms by which bacteria colonize and survive on leaf surfaces remain largely unknown (Burch *et al.*, 2016). Healthy epidermal plant cells constantly leak organic and inorganic molecules, such as amino acids, sugars, and various salts to their surfaces; this may facilitate the colonization of microflora by interacting with the plant phyllosphere (Knief *et al.*, 2012; Burch *et al.*, 2016). Furthermore, methanol released from stomata through cell expansion attracts several bacterial strains (Nemecek-Marshall *et al.*, 1995). The genus *Methylobacterium* is composed of pink-pigmented facultative methylotrophs (PPFMs) that belong to the α-Proteobacteria class, and use methanol as the sole carbon and energy source (Abanda-Nkpwatt *et al.*, 2006). *Methylobacterium* spp. express several genes related to methylotrophy during the colonization of plants, such as rice, citrus, peanut, lettuce, tobacco, and strawberry (Lee *et al.*, 2006; Knief *et al.*, 2012; Rastogi *et al.*, 2012; Mwajita *et al.*, 2013). Several isolates of the genus *Methylobacterium* have been reported to enhance plant growth by producing phytohormones, such as auxin and cytokinins, fixing atmospheric nitrogen, and utilizing 1-aminocyclopropane-1-carbonxylate (ACC) deaminase activity to reduce stress-related ethylene production. The dominant colonization of *Methylobacterium* spp. facilitates healthy growth of the rice plant by protecting it from infection and colonization by other potential pathogens (Lee *et al.*, 2006; Kwak *et al.*, 2014; Madhaiyan *et al.*, 2015).

Vietnam is the fifth largest rice-producing country in the world (FAO 2017), and rice export plays a pivotal part in its economic development. The Mekong Delta is the major rice-producing area in Vietnam and one of the biggest globally. Thus, healthy and productive rice cultivation is of great importance. Although the composition of the bacterial community plays an important role in the healthy growth of rice plants to produce high yields of rice, microbial diversity on the rice plant phyllosphere and rhizosphere in the Mekong delta has not yet been reported. The present study was undertaken to reveal the genetic diversity of *Methylobacterium* spp. in the rice of the Vietnamese Mekong delta and examine their effects on rice plant growth.

To isolate *Methylobacterium* spp. from rice plants, we collected rice samples at the flowering stage (60–70‍ ‍d after sowing) from six provinces–Can Tho, Vinh Long, Tien Giang, Ho Chi Minh, Dong Nai, and Khanh Hoa. All rice plants sampled were *Oryza sativa* cultivar OM5451.

The number of samples and sampling sites are shown in Fig. 1. Fresh samples (10 g) of rice roots and shoots were separately homogenized with 90 mL sterilized 0.85% NaCl solution and plated onto Murashige and Skoog (MS) medium supplemented with 0.5% (v/v) methanol and 10‍ ‍μg‍ ‍mL^–1^ cycloheximide. Plates were incubated at 28°C for 7 to 8 d. Single, well-isolated, and pinkish colonies from the isolation medium were sub-cultured, purified, and maintained in glycerol stocks. Genomic DNA of the isolates was extracted using the Wizard Genomic DNA purification kit (Promega). The 16S rRNA gene was amplified by PCR using the primers 16AF (5′-AACTGAAGAGTTTGATCMTGGCTCAG-3′) and 1492R (5′-TACGGYTACCTTGTTACGACTT-3′). The 16S rRNA gene sequence was searched for homology by BLAST (Basic Local Alignment Search Tool) at the NCBI (National Center for Biotechnology Information) website. Sequence alignment was performed using MEGA software ver 7.0 (Kumar *et al.*, 2016). Phylogenetic trees were constructed using the neighbor-joining method.

Rice (*O. sativa* Nipponbare) and lettuce (*Lactuca sativa* L. cv. *Legacy*) were used to evaluate the effects of *Methylobacterium* spp. on plant growth. Rice seeds were sterilized by soaking in 70% ethanol for 1 min and 5% sodium hypochlorite with 0.02% Tween 20 for 10 min, and subsequently washed five times with sterile water. Lettuce seeds were sterilized by soaking in 1% sodium hypochlorite solution for 5 min followed by 70% ethanol for 3 min, and subsequently washed five times with sterile water. *Methylobacterium* isolates were grown in 5 mL CMS medium (MS medium with sucrose 30 g L^–1^, peptone 2‍ ‍g‍ ‍L^–1^, and tryptone 2 g L^–1^) for 4 d, after which they were washed twice, and bacterial cells were diluted to a final concentration of 10^4^ cfu mL^–1^ in sterile water. Surface-sterilized seeds were soaked overnight in a bacterial suspension. Control seeds were treated with sterile distilled water without the bacterial culture. Seeds (12 for rice and 5 for lettuce) were sown in a 300-mL plant box (CUL-JAR300; Iwaki) containing 30 g sterile vermiculite (Hirukon S, Hiruishi Kagaku Kogyo) and watered with 25% MS medium without sucrose. Three replicates for each treatment condition were used in all plant inoculation tests. Plants were grown in a growth chamber at 25°C (8 h dark/16 h light cycle) for 14 d. Seedling growth was evaluated by measuring leaf and root lengths and the fresh weight of leaves.

Sixty-one putative *Methylobacterium* strains were isolated from 24 rice plants collected from paddy fields across six provinces (Fig. 1). All 61 strains were identified by 16S rRNA gene sequencing and categorized into seven species (Fig. S1). The largest cluster belonged to *M. radiotolerans* (29/61 isolates), followed by *M. salsuginis* (17/61 isolates). Of the seven species of the genus *Methylobacterium* isolated from rice plants in Vietnam, five (*M. komagatae*, *M. salsuginis*, *M. indicum*, *M. radiotolerans*, and *M. oryzae*) were isolated from rice cultivated in other parts of the world (Madhaiyan *et al.*, 2006; Knief *et al.*, 2012; Kwak *et al.*, 2014). *M. rhodium* was previously found in water samples (Green and Bosfield, 1983), but has never been isolated from rice. Furthermore, one of the isolates, HP16.3 was phylogenetically ambiguous and located between three species, *M. platani*, *M. aquaticum*, and *M. tarhaniae*, based on 16S rRNA gene phylogeny.

In the present study, *Methylobacterium* spp. were more frequently isolated from the shoots (80%) than from the roots (20%) (Fig. 2A and Table S1). Previous studies on the isolation of *Methylobacterium* spp. from rice plants demonstrated that the distribution of *Methylobacterium* spp. was dependent on the area of colonization (Lee *et al.*, 2006; Sun *et al.*, 2008; Knief *et al.*, 2012). Metagenomics and metaproteomics analyses previously revealed that α-Proteobacterium, particularly *Rhizobium* and *Methylobacterium*, were more frequently found in the rice phyllosphere than in the rice rhizosphere (Knief *et al.*, 2012). Similarly, Sun *et al.* (2008) used 16S rDNA cloning, an amplified ribosomal DNA restriction analysis, and sequence homology comparisons to demonstrate that *Methylobacterium* spp. are present at a low frequency in rice roots (Sun *et al.*, 2008); our results are consistent with these findings. Interestingly, 53% of isolates found in the rice phyllosphere belonged to *M. radiotolerans* (Fig. 2A). The upper part of plants (the phyllosphere) is generally considered to be a harsh environment for microbial colonization due to stress from water, UV irradiation, temperature changes, and limited nutrient availability (Lee *et al.*, 2006; Maliti *et al.*, 2007). One possibility is that *M. radiotolerans* isolates in the present study exhibit high-stress tolerance, facilitating their adaptation and survival in extreme environments, such as phyllosphere habitats. The predominant factors that transfer these bacteria into plants are still being debated. Romanovskaya *et al.* (2001) proposed that under natural conditions, *Methylobacterium* strains colonize the plant leaves, being transferred to their surface by air with soil particles (Romanovskaya *et al.*, 2001). Furthermore, previous studies reported that *Methylobacterium* spp. were transferred from plants seeds to leaves (Omer *et al.*, 2004; Madhaiyan *et al.*, 2006). The present results indicate that environmental factors, such as temperature, air, and UV light, play a pivotal role in forming the community structure of *Methylobacterium* inhabitants.

We assessed the relationship between geographic locations and specific *Methylobacterium* species. We found that 68% isolates belonged to *M. radiotolerans* isolated from three provinces. Ho Chi Minh, Dong Nai, and Khanh Hoa—in the northern region of the Mekong delta. However, the southern region of the Mekong delta, consisting of Tien Giang, Vinh Long, and Can Tho province, was abundant in *M. salsuginis* (71%) (Fig. 2B). The southern region of the Mekong delta has recently faced the threat of saltwater intrusion, which may damage agricultural activities, including rice cultivation. Since *M. salsuginis* was initially isolated from the South China sea and has been well characterized, we suggest that a location with a high salt concentration in the soil influences *Methylobacterium* spp. distribution in the Mekong delta (Wang *et al.*, 2007; Vu *et al.*, 2018). Similarly, Knief *et al.* (2012) examined the effects of the host plant, location, time, and other bacterial communities on the composition and population size of *Methylobacterium*. Their findings demonstrated that the location-specific factor exerted a stronger impact than the host plant-specific factor (Knief *et al.*, 2012). Our results are consistent with these findings indicating that specific locations influence the distribution of *Methylobacterium* spp.

*Methylobacterium* spp. promote seed germination and growth in several plants (Lee *et al.*, 2006). Inoculation tests revealed that most *Methylobacterium* spp. isolated from the Mekong delta did not significantly affect rice growth (data not shown). However, some isolates exerted growth-inducing effects on rice plants (Table 1). Inoculations with *Methylobacterium* strains HP18.2, HP19.2, DR25.3, CP40.1, and VR43.1 enhanced plant growth and increased plant biomass over those obtained using an un-inoculated control. These isolates increased the root length from 7 to 14% and leaf weight up to 8% over those of the un-inoculated control. No significant difference was observed between these strains; however, some strains, such as HP19.2 and CP40.1, showed increased root and leaf lengths or leaf weights. Lee *et al.* (2006) reported that the inoculation of *Methylobacterium* strain CBMB20 with rice increased the rate of seed germination, seedling parameters, and seedling biomass by producing phytohormones, such as trans-zeatin riboside and indole-3-acetic acid. Other *Methylobacterium* strains Q4 and Q5 significantly stimulated the growth of two rice cultivars, japonica cultivar CR76 and indica cultivar A301 on four parameters—root development, leaf growth, and stem and biomass productivities (Maliti *et al.*, 2007). Isolates of *Methylobacterium* spp. also effectively promoted the growth of various seed plants (Abanda-Nkpwatt *et al.*, 2006).

*Methylobacterium* isolates, including those that promoted the growth of rice plants, were tested on lettuce plants. As shown in Table 1, isolates promoted the growth of lettuce plants; leaf length increased from 5 to 9% and leaf weight up to 11%. Tani *et al.* (2012) reported that *Methylobacterium* strain 22A isolated from a liquid culture of the moss *R. japonicum* promoted the growth of the tested seed plants, including *Arabidopsis*, *Nicotiana*, and *Hordeum* (Tani *et al.*, 2012). In other studies, *M. extorquens* strain ME4 was shown to promote the growth of plants, such as *Lycopersicon*, *Fragaria*, *Sinapis*, and *Nicotiana*, but exerted no effect on seed germination in *Triticum*, *Daucus*, maize, *Hordeum*, and *Pisum* (Maliti *et al.*, 2007). Our results are consistent with the findings demonstrating the positive effects of *Methylobacterium* spp. on plant growth.

Notably, three isolates belonging to *M. indicum* (VP43.2, DP28.3, and DP28.4) did not enhance plant growth, but exerted a negative effect on rice seedlings (Fig. 3). Following a 5-day inoculation with VP43.2, DP28.3, and DP28.4 isolates, rice leaves turned white. During this period, the growth of plant seedlings was retarded and plants died within 2‍ ‍weeks. *Methylobacterium* spp. are related to more than 70 plant species. However, the mechanisms by which *Methylobacterium* spp., including *M. indicum*, exert a negative effect on plant growth remain unclear (Chaudhry *et al.*, 2016). Thus, this is the first study to suggest that *Methylobacterium* strains cause disease-like symptoms similar to plant pathogens. The “bleaching” symptom is completely different from other disease symptoms. The “cold damage” symptom has been described as a white band on the leaf blade during exposure to cold temperatures (Da Cruz *et al.*, 2013). “Bacterial leaf blight” caused by *Xanthomonas oryzae* was previously characterized by elongated lesions near the leaf tip or margin that are several inches long, which turn white to yellow and then gray (Sharma *et al.*, 2017). The “bleaching” symptom caused by *Methylobacterium* strains changed the color of rice plant leaves from green to yellow, and gradually to white; infected rice plants ultimately died. Further studies are needed to identify the pathogenic genes in these isolates and elucidate the physiological mechanisms underlying this novel disease in rice.

In conclusion, the present study revealed the genetic diversity of rice-associated *Methylobacterium* spp. in the Vietnamese Mekong delta. Sixty-one isolates belonging to seven species were similar to those previously isolated from rice plants; however, their genetic diversity was presumed to be influenced by unique environmental factors, such as saline soil in the Mekong delta. Most of the isolates tested exerted no or weak plant growth-promoting effects, suggesting that these *Methylobacterium* spp. improves the health of rice plants by their influence on host resistance. Of interest, three isolates showed disease symptoms in rice, which is the first evidence of *Methylobacterium* functioning as a plant pathogen. These results reveal a complex diversity of rice-associated *Methylobacterium* in the Mekong delta and suggest that healthy and productive rice cultivation requires a well-balanced microbial community, particularly *Methylobacterium* spp.

## Supplementary Material

Supplementary Material

## Figures and Tables

**Fig. 1.**
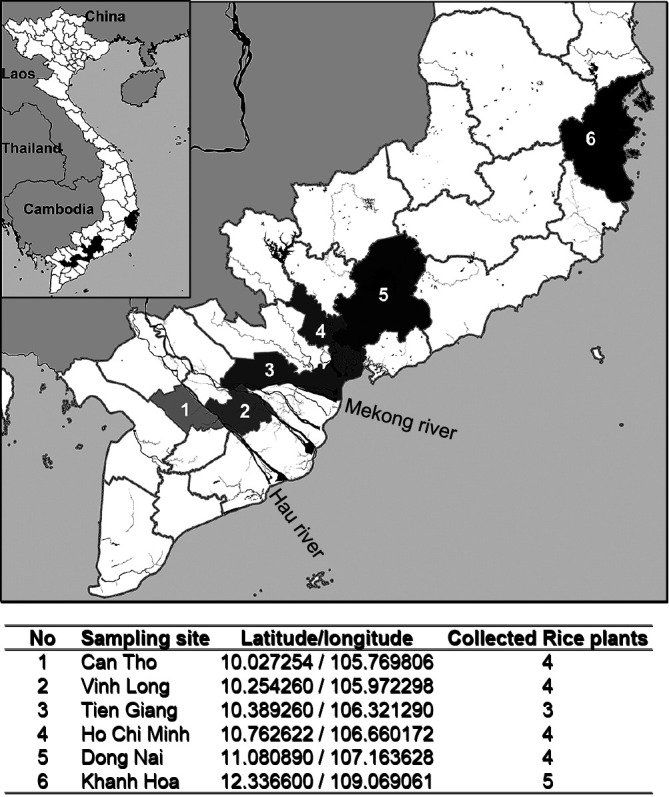
Sampling sites of rice samples. Rice samples were collected from six sites indicated by different colors.

**Fig. 2.**
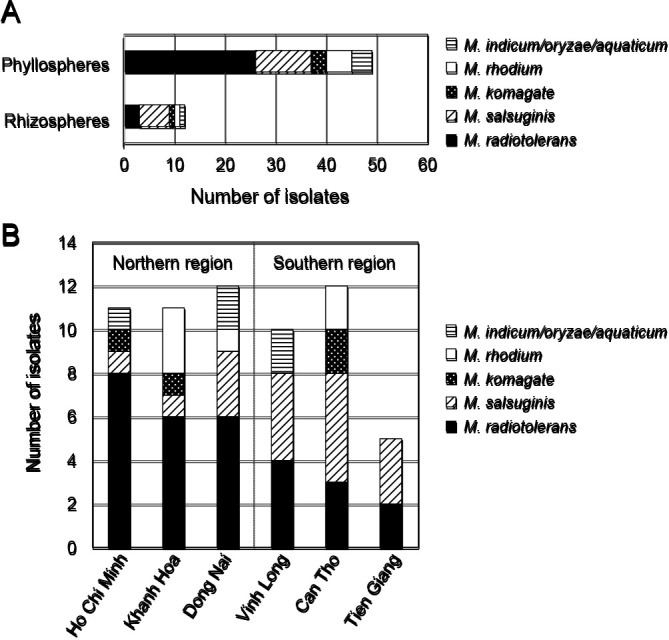
Distribution of *Methylobacterium* spp. according to plant locations and isolation sites. (A) The percentages of each species isolated from the phyllosphere and rhizosphere are as follows: *M. radiotolerans* (90%, 10%), *M. salsuginis* (65%, 35%), *M. komagatae* (75%, 25%), *M. rhodium* (83%, 17%), and *M. indicum/oryzae/aquaticum* (80%, 20%). (B) The percentages of each species isolated from the Northern region (Dong Nai, Ho Chi Minh, and Khanh Hoa) and Southern region (Tien Giang, Vinh Long, and Can Tho) of the Mekong delta are as follows: *M. radiotolerans* (68%, 32%), *M. salsuginis* (29%, 71%), *M. komagatae* (50%, 50%), *M. rhodium* (66%, 33%), and *M. indicum/oryzae/aquaticum* (60%, 40%).

**Fig. 3.**
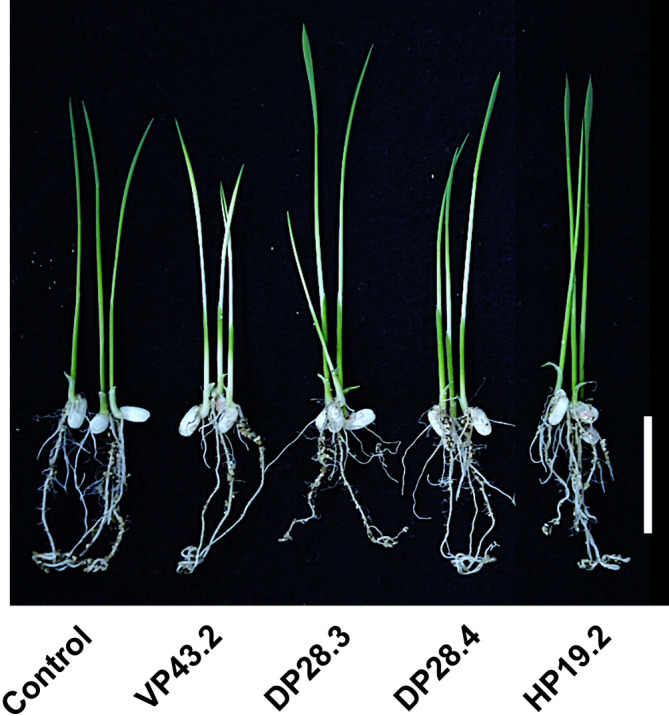
Bleaching symptom of rice seedlings. This image was acquired following a 5-day inoculation with VP43.2, DP28.3, and DP28.4 isolates. Un-inoculated seedlings were used as the control and seedlings inoculated with HP19.2 did not exhibit any bleaching symptom. Scale bar=2 cm.

**Table 1.** Effects of selected *Methylobacterium* isolates on rice and lettuce growth.

Plant parameters	Treatments (isolates)						
	Un-inoculated	HP18.2	HP19.2	DR25.3	DP26.3	CP40.1	VR43.1
Length of rice roots (cm)	10.4±1.2^c^	10.9±1.3^bc^	11.5±1.3^ab^	11.4±1.2^ab^	10.4±1.4^c^	11.2±1.2^abc^	11.9±1.4^a^
Length of rice leaves (cm)	23.0±1.9^b^	23.9±2.4^ab^	23.6±2.2^ab^	23.5±2.5^ab^	23.3±2.2^ab^	24.6±1.1^a^	23.0±2.5^b^
Weight of rice leaves (g)	0.124±0.008^c^	0.136±0.022^a^	0.134±0.007^ab^	0.124±0.016^bc^	0.126±0.010^bc^	0.130±0.015^abc^	0.126±0.018^bc^
Length of lettuce leaves (cm)	5.6±0.2^b^	6.1±0.3^a^	5.9±0.3^a^	5.9±0.2^a^	6.0±0.3^a^	5.8±0.3^ab^	5.8±0.4^ab^
Weight of lettuce leaves (g)	0.115±0.011^b^	0.125±0.011^a^	0.127±0.008^a^	0.121±0.010^ab^	0.123±0.011^ab^	0.117±0.005^ab^	0.117±0.005^ab^

Data represent means±SD (*n*=36 for rice and *n*=15 for lettuce). Means followed by different letters are significantly different at the 5% level (ANOVA with Tukey’s test).
