# Supplementary material for: Diversity of Methylobacterium spp. in the Rice of the Vietnamese Mekong Delta
Source: Microbes Environ. 2020 Jan 23;35(1):ME19111. doi: 10.1264/jsme2.ME19111 (PMC7104282; doi:10.1264/jsme2.ME19111)
Supplement: Supplementary file 1 — Supplementary Material [file 35_19111_s1.pdf]

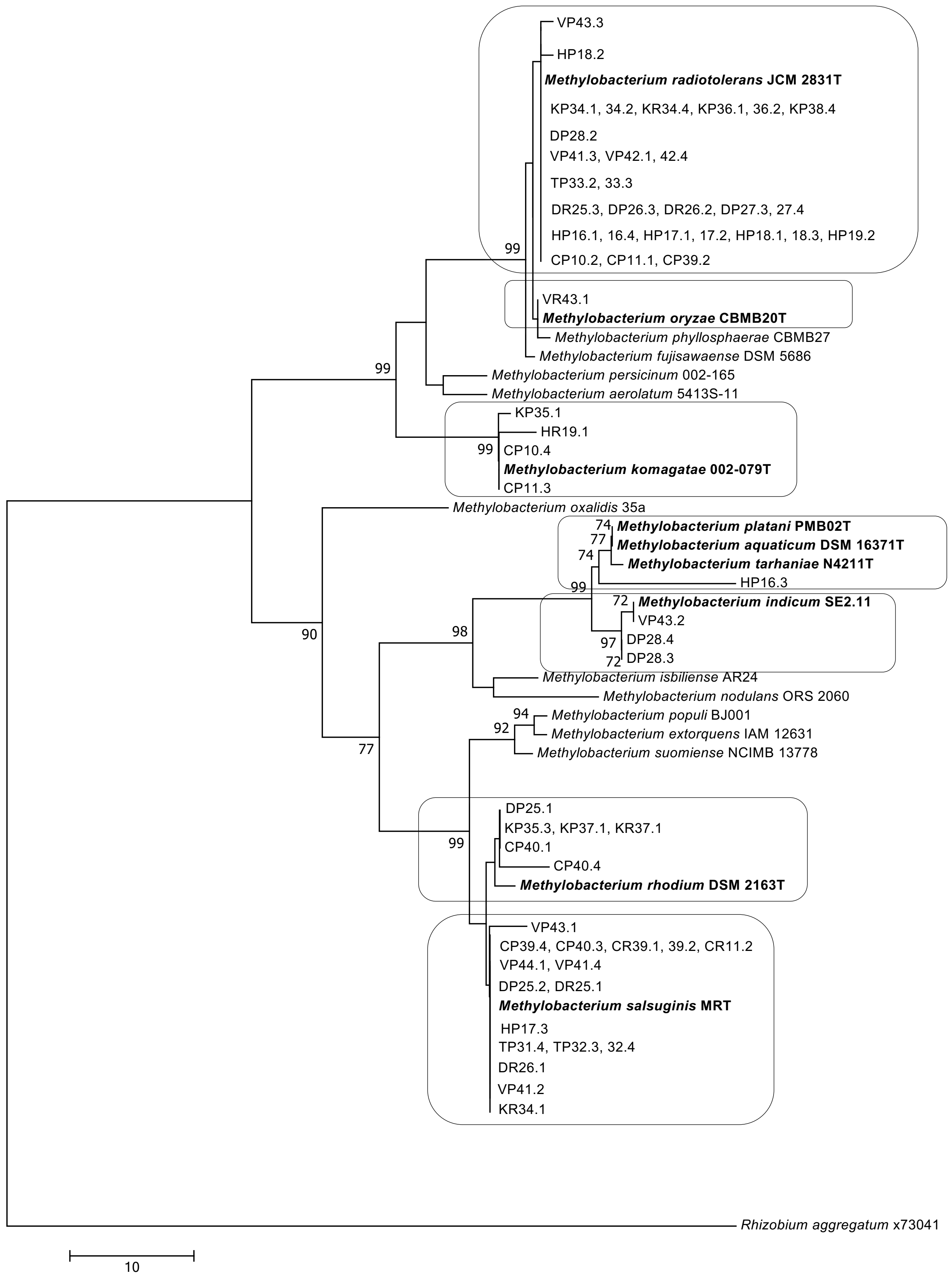

Figure S1. Figure S1. Phylogenetic tree for 16S rRNA gene sequences of isolates. Phylogenetic tree was constructed using about 1300 bp nucleotide sequences of 16S rRNA gene from 61 isolates (Table S1) and the type strains of each specie belonging to genus *Methylobacterium*. Bootstrap value were shown as percentage of 1000 replicates.

(Khoa et al., 2019)

Table S1. ***Methylobacterium*** **strains isolated in this study.**

Region	Rice Samples	Strain name[a]	Isolated plant part	Species	Accession number
**Can Tho**	1	CP10.2	Phyllospheres	*M. radiotolerans*	MK968394
		CP10.4	Phyllospheres	*M. komagatae*	MK968393
	2	CP11.3	Phyllospheres	*M. komagatae*	MK968416
		CP11.1	Phyllospheres	*M. radiotolerans*	MK968417
		CR11.2	Rhizospheres	*M. salsuginis*	MK968376
	3	CP39.2	Phyllospheres	*M. radiotolerans*	MK968428
		CP39.4	Phyllospheres	*M. salsuginis*	MK968372
		CR39.1	Rhizospheres	*M. salsuginis*	MK968373
		CR39.2	Rhizospheres	*M. salsuginis*	MK968374
	4	CP40.1*	Phyllospheres	*M. rhodium*	MK968386
		CP40.3	Phyllospheres	*M. salsuginis*	MK968375
		CP40.4	Phyllospheres	*M. rhodium*	MK968426
**Vinh Long**	5	VP41.2	Phyllospheres	*M. salsuginis*	MK968430
		VP41.3	Phyllospheres	*M. radiotolerans*	MK968411
		VP41.4	Phyllospheres	*M. salsuginis*	MK968378
	6	VP42.1	Phyllospheres	*M. radiotolerans*	MK968418
		VP42.4	Phyllospheres	*M. radiotolerans*	MK968413
	7	VP43.2**	Phyllospheres	*M. indicum*	MK968429
		VP43.1	Phyllospheres	*M. salsuginis*	MK968427
		VP43.3	Phyllospheres	*M. radiotolerans*	MK968422
		VR43.1*	Rhizospheres	*M. oryzea*	MK968414
	8	VP44.1	Phyllospheres	*M. salsuginis*	MK968377
**Tien Giang**	9	TP31.4	Phyllospheres	*M. salsuginis*	MK968383
	10	TP32.3	Phyllospheres	*M. salsuginis*	MK968384
		TP32.4	Phyllospheres	*M. salsuginis*	MK968385
	11	TP33.2	Phyllospheres	*M. radiotolerans*	MK968402
		TP33.3	Phyllospheres	*M. radiotolerans*	MK968401
**Ho Chi Minh**	12	HP16.1	Phyllospheres	*M. radiotolerans*	MK968400
		HP16.3	Phyllospheres	*M. aquaticum*	MK968390
		HP16.4	Phyllospheres	*M. radiotolerans*	MK968399

	13	HP17.1	Phyllospheres	*M. radiotolerans*	MK968397
		HP17.2	Phyllospheres	*M. radiotolerans*	MK968398
		HP17.3	Phyllospheres	*M. salsuginis*	MK968382
	14	HP18.1	Phyllospheres	*M. radiotolerans*	MK968396
		HP18.2*	Phyllospheres	*M. radiotolerans*	MK968425
		HP18.3	Phyllospheres	*M. radiotolerans*	MK968395
	15	HP19.2*	Phyllospheres	*M. radiotolerans*	MK968421
		HR19.1	Rhizospheres	*M. komagatae*	MK968392
**Dong Nai**	16	DP25.1	Phyllospheres	*M. rhodium*	MK968389
		DP25.2	Phyllospheres	*M. salsuginis*	MK968379
		DR25.1	Rhizospheres	*M. salsuginis*	MK968380
		DR25.3*	Rhizospheres	*M. radiotolerans*	MK968410
	17	DP26.3*	Phyllospheres	*M. radiotolerans*	MK968423
		DR26.2	Rhizospheres	*M. radiotolerans*	MK968409
		DR26.1	Rhizospheres	*M. salsuginis*	MK968424
	18	DP27.3	Phyllospheres	*M. radiotolerans*	MK968408
		DP27.4	Phyllospheres	*M. radiotolerans*	MK968407
	19	DP28.2	Phyllospheres	*M. radiotolerans*	MK968412
		DP28.3**	Phyllospheres	*M. indicum*	MK968432
		DP28.4**	Phyllospheres	*M. indicum*	MK968431
**Khanh Hoa**	20	KP34.1	Phyllospheres	*M. radiotolerans*	MK968405
		KP34.2	Phyllospheres	*M. radiotolerans*	MK968415
		KR34.1	Rhizospheres	*M. salsuginis*	MK968381
		KR34.4	Rhizospheres	*M. radiotolerans*	MK968419
	21	KP35.1	Phyllospheres	*M. komagatae*	MK968391
		KP35.3	Phyllospheres	*M. rhodium*	MK968420
	22	KP36.1	Phyllospheres	*M. radiotolerans*	MK968403
		KP36.2	Phyllospheres	*M. radiotolerans*	MK968404
	23	KP37.1	Phyllospheres	*M. rhodium*	MK968387
		KR37.1	Rhizospheres	*M. rhodium*	MK968388
	24	KP38.4	Phyllospheres	*M. radiotolerans*	MK968406

[a]Strains with a single asterisk (*) indicate those exhibited plant growth stimulation and those with a double asterisks (**) exhibited negative effect on rice plant in the inoculation test.
